# Temperature Modeling of AISI 1045 Steel during Surface Hardening Processes

**DOI:** 10.3390/ma11101815

**Published:** 2018-09-25

**Authors:** Tsung-Pin Hung, Hao-En Shi, Jao-Hwa Kuang

**Affiliations:** 1 Department of Mechanical Engineering, Cheng Shiu University, Kaohsiung 840, Taiwan; 2 Department of Mechanical and Electro-Mechanical Engineering, National Sun Yat-sen University, Kaohsiung 804, Taiwan; tsungpin@gmail.com (H.-E.S.); kuang@faculty.nsysu.edu.tw (J.-H.K.)

**Keywords:** self-quenching effect, laser scanning, heat treatment

## Abstract

A Coupled thermo-mechanical finite element model was employed to simulate the possible effects of varying laser scanning parameters on the surface hardening process for AISI 1045 and AISI 4140 steels. We took advantage of the high-power density of laser beams to heat the surface of workpieces quickly to achieve self-quenching effects. The finite element model, along with the temperature-dependent material properties, was applied to characterize the possible quenching and tempering effects during single-track laser surface heat treatment. We verified the accuracy of the proposed model through experiments. The effects of laser surface hardening parameters, such as power variation, scanning speed, and laser spot size, on the surface temperature distribution, hardening width, and hardening depth variations during the single-track surface laser treatment process, were investigated using the proposed model. The analysis results show that laser power and scanning speed are the key parameters that affect the hardening of the material. The numerical results reveal that the proposed finite element model is able to simulate the laser surface heat treatment process and tempering effect of steel.

## 1. Introduction

In 1868, Russian metallurgist D. K. Chernov discovered that the heating and quenching of steel alters its internal structure and thus proposed the iron-carbon phase diagram [1]. A variety of heat treatment techniques have been developed for over a hundred years since this discovery, including, for example, furnace-based heat treatment, induction heating-based heat treatment, flame hardening, and chemical heat treatment. Over the last 50 years alone, scientists around the world invented several novel heat treatment techniques, including vacuum heat treatment, austenite grain refinement, and heat treatment methods for the control of martensitic and bainitic structures. Laser surface heat treatment and laser surface cladding are among the most well-known heat treatment methods to have emerged over the past decade.

As the laser energy absorption rate of a material depends on the wavelength of the laser, at present, the most popular laser sources in the market are direct-diode lasers, carbon dioxide (CO_2_) lasers, neodymium-doped yttrium aluminum garnet (Nd:YAG) lasers, argon lasers, and ruby lasers. Direct-diode lasers and Nd:YAG lasers are commonly used in surface heat treatments because these laser sources typically have high power densities and the energy absorption rates of steel for these lasers are very high. In particular, the energy absorption rates of AISI 1045 steel for 10.6-μm Nd:YAG and CO_2_ lasers are 8% and 40%, respectively. It has thus been shown that Nd:YAG lasers have an excellent energy consumption-to-cost ratio, which is why we chose to use an Nd:YAG laser in this study [2].

In 2002, Ganeev experimentally investigated the effectiveness of low-power CO_2_ lasers in the multi-track hardening of a steel surface in terms of the hardness of the processed surface and hardening depth [3]. In 2006, Skvarenina and Shin used experimental and numerical approaches to study surface hardness and hardening depth in AISI 1536 steels that were processed using a variety of laser quenching parameters. In addition, they also calculated the laser energy absorption rate of AISI 1536 [4]. In the following year, Lakhkar et al. employed numerical analysis to investigate how the back-tempered zone affects hardening depth and surface hardness during multi-track laser hardening in AISI 4140 alloy steel [5]. In 2008, Bailey et al. employed experimental and numerical approaches to study the residual stresses of laser-hardened AISI 4140 alloy steel workpieces [6]. In the year afterwards, Farrahi and Sistaninia employed a variety of laser scanning patterns to investigate their effects on the laser surface hardening of AISI 1036 alloy steel [7].

In 2013, Lambiase et al. combined analysis models with a neural network to investigate the temperature distributions and transient temperature changes associated with laser surface hardening processes and to predict the hardness of laser-hardened surfaces [8]. In the same year, Babic et al. conducted an experimental study on laser surface hardening using a CO_2_ laser installed on a robotic arm, in which they investigated the effects of laser overlap on surface hardness [9]. El-Batahgy et al. used experimental and numerical approaches to investigate the laser surface hardening of AISI M2 high-speed tool steel. The maximum hardness and wear resistance of the laser-hardened specimens were 23% and 30% higher than that of conventionally heat-treated specimens, respectively. In addition, their numerical simulations were in good agreement with their experimental results, which proves that numerical simulations can be used to predict surface temperature, surface hardness, and hardening depth for a wide range of laser processing parameters [10]. Heitkemper et al. investigated the mechanical and chemical properties of martensitic high-nitrogen tool steels that had undergone laser surface heat treatment, and they showed that laser heat treatment improved the wear resistance, fatigue resistance, and corrosion resistance of these steels [11]. In 2014, Ki et al. proposed a heat-sink assisted laser hardening process and compared the results of laser hardening with four different heat sink configurations (using either stainless steel, steel, or copper heat sinks or no heat sink at all). They showed that the use of heat sinks improves the hardenability of steel sheets and their hardness values [12]. In the same year, Orazi et al. proposed a laser surface hardening method for cylindrical workpieces. This method produces a uniform and deep hardened layer across the entire work piece surface without introducing a tempered zone [13]. Kostov et al. investigated the effects of process atmosphere on the microstructures and residual stresses that originate from the laser surface hardening of AISI 4140 steel. They showed that an evacuated or helium-filled atmosphere prevents the formation of an oxide layer, which is beneficial for laser surface hardening. However, the use of a helium atmosphere widens the hardened zone and compressively stressed region [14]. Li et al. experimentally investigated the use of high-power diode lasers (HPDLs) and CO_2_ lasers for the laser surface hardening of AISI 1045 medium-carbon steel. The HPDLs had a rectangular beam spot with a uniform energy distribution, while the CO_2_ lasers had a circular beam spot with a Gaussian energy distribution. Their results show that HPDLs are highly effective for surface hardening processes; surface melting was not observed on the HPDL-treated specimen and the hardness of the hardened layer was practically constant across its entire profile. Surface melting was observed in the CO_2_ laser-treated specimen, which may be due to the laser’s Gaussian energy distribution. Furthermore, the hardness of the hardened layer in this specimen decreased with depth; the effectiveness of CO_2_ lasers in surface hardening processes was therefore deemed less than ideal [15]. In 2016, Liverani et al. used numerical and experimental approaches to construct a numerical model for the prediction of residual stress distributions and hardening depth in laser-hardened surfaces [16]. In the same year, Sarkar et al. investigated the laser surface hardening of low-carbon steel (0.05% and 0.07% C) using high-power Yb-fiber lasers. They found that the average Vickers hardness of 0.05% C steel was increased from 120 HV to 217 HV, while the average Vickers hardness of 0.07% C steel was increased from 160 HV to 280 HV [17]. Sato et al. proved that laser surface hardening technology is widely applicable for sintered parts [17]. Bouquet et al. developed an integrated laser hardening system that is also equipped with an ultrasonic-assisted grinding system. This system was then used to conduct a laser hardening study on C45 medium-carbon steel. In that study, numerical and experimental methods were used to study the relationship between hardening depth, hardness, temperature, and material melting when laser surface hardening was performed with different process parameters [18]. This integrated laser hardening system is also able to perform multiple machining processes on a single workpiece, which reduces the time used for workpiece clamping and re-alignment, thus improving production efficiency [19]. In 2017, Farshidianfar et al. experimentally investigated the effects of laser scanning speeds and laser power on the microstructure, surface hardness, and hardening depth of AISI low-carbon steel and also how these properties correlate with the aforementioned laser parameters [20]. In the same year, Guarinoa et al. performed an experiment in which diode lasers were used to perform surface hardening treatments on AISI 1040 medium-carbon steel. In this work, they investigated the effectiveness of laser heat treatments for hardening material surfaces and the effects of laser process parameters on fatigue life [21].

As tool steels are typically not prone to quench cracking and have high levels of strength, ductility, and heat treatment hardness, heat treatments are usually more effective for tool steels than structural steels. Consequently, tool steels are usually used to fabricate important components, such as crankshafts, linkages, gears, and cams. It is very challenging to control temperatures in a stable and reliable manner when hardening treatments are being performed in local sections of a large surface. To address this issue, we have proposed a finite element analysis model in this work and experimentally validated its accuracy. The numerical analysis model was then used to numerically simulate how laser scanning speed, laser power, and laser spot size affect hardening depth, hardening width, maximum laser spot temperature, and the back-tempering effect in AISI 1045 and AISI 4140 steels. We hope that the predictions of the finite element analysis model enable users to derive optimal parameters for laser surface heat treatments.

## 2. Finite Element Analysis Modeling 

**Figure 1:**
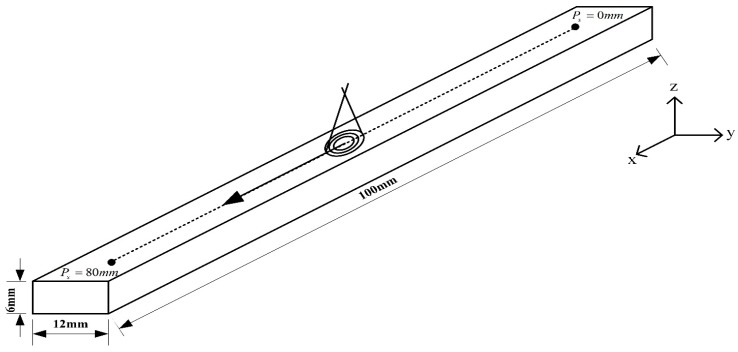
Proposed laser heat treatment model.

The numerical analyses of this study were performed using the thermo-elastic-plastic models of the commercial MSC Marc software suite for finite element analysis. Coupled thermo-mechanical analysis was used to improve the physical accuracy of our calculations. During each iteration of the simulation, the actual temperature distribution of the model is acquired and the corresponding strains and stresses of the model are also calculated. This ensures that the model will, at any point in time, satisfy all equilibrium equations and convergence conditions. This method of numerical analysis produces results that are more accurate than uncoupled thermomechanical methods. To ensure a reasonable level of computational efficiency, a partial workpiece was selected, as shown in Figure 1.

**Figure 2:**
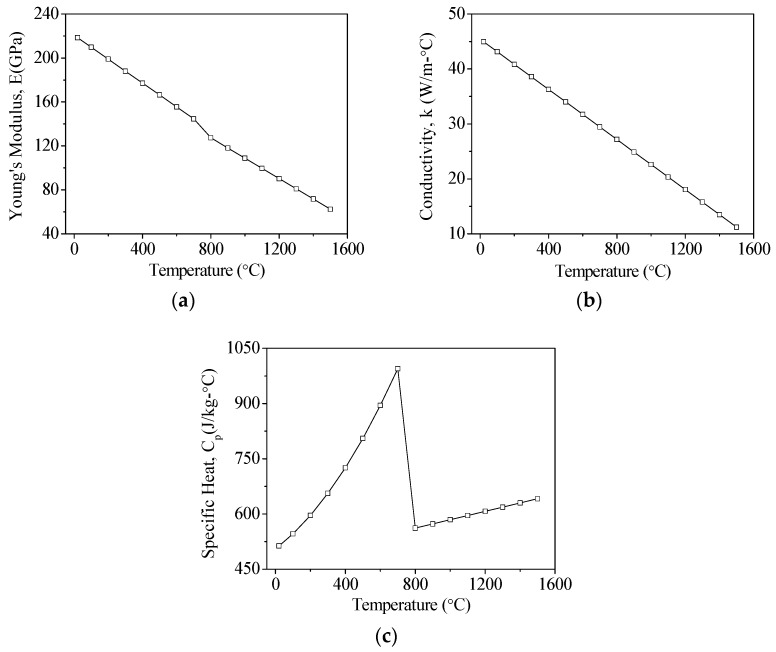
The temperature depenent material properties of AISI 1045. (**a**) Young’s modulus; (**b**) Coefficient of thermal conductivity; and (**c**) Specific heat.

**Figure 3:**
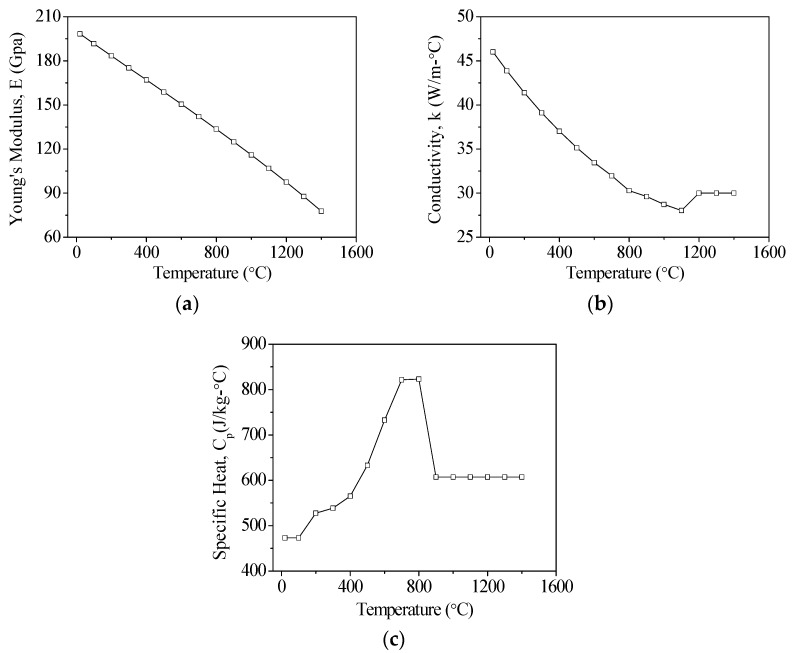
The temperature dependent material properties of AISI 4140. (**a**) Young’s modulus; (**b**) Coefficient of thermal conductivity; and (**c**) Specific heat.

The mechanical properties of AISI 1045 [22] and AISI 4140 [23,24] steels are shown in Table 1. The changes in Young’s modulus, conductivity, and specific heat of these materials for changes in temperatures are shown in Figure 2 and Figure 3 Based on the existing literature, the hardening and melting temperatures of AISI 1045 are 760 °C and 1520 °C, while the hardening and melting temperatures of AISI 4140 are 850 °C and 1410 °C, respectively. These temperature ranges were used to determine whether a material was successfully hardened. On the boundary condition setting, it is assumed that the laser energy is focused on the surface of the material. The initial temperature of the material was set to 25 °C, and the temperature of the material was reduced to room temperature in consideration of the natural heat convection effect of the air, and the heat convection coefficient was assumed to be 12.6 W/m^2^·°C. 

### 2.1. Construction of the Thermal Source Models

**Figure 4:**
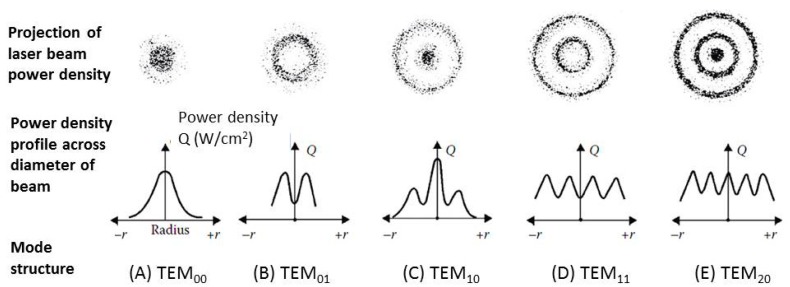
Heat source distributions of various transverse electromagnetic modes (TEM) [25].

There are many ways to assume the distribution of heat sources. For example, Lostado Lorza used the Double Ellipsoidal Weld Flux setting to describe the GMAW (Gas Matal Arc Welding) heat source distribution [25,26]. The laser source used in this study was an Nd:YAG laser, and the heat source distribution of the laser was set to transverse electromagnetic mode 00 (*TEM*_00_) [27], which is one of the most common laser modes, as shown in Figure 4. This distribution is somewhat different from that of conventional welding heat sources, which usually have a bi-elliptical distribution. The *TEM*_00_ distribution is characterized by high power densities at the center of the light source, which gradually decreases as the distance from the center increases; this is typical of Gaussian distributions.

The power density of a *TEM*_00_ laser may be expressed as:

(1) $$E_{e}(r_{e})=P_{i}\cdot \Phi (r_{e}),$$

where *r_e_* is the distance from the center point of the laser spot (laser focal spot radius is 0.5 mm), *P_i_* is the magnitude of the laser power, and Φ is the Gaussian distribution function.

The one-dimensional Gaussian distribution function is:

(2) $$\Phi (r_{e})=\frac{1}{\sqrt{2\pi }s}\exp(-\frac{r_{e}{}^{2}}{2s^{2}}),$$

where *s* is the standard deviation.

As the Gaussian distribution is a probability function, it satisfies:

(3) $$\int {}_{0}^{0.5}\Phi (r_{e})dr_{e}=1,$$

A two-dimensional Gaussian distribution may be obtained by rotating the 2D Gaussian distribution by 360°, which gives:

(4) $$\Phi (r_{e})=\frac{1}{2\pi s^{2}}\exp(-\frac{r_{e}{}^{2}}{2s^{2}}).$$

This also satisfies:

(5) $$\int {}_{0}^{2\pi }\Phi (r_{e})r_{e}dr_{e}d\theta =1.$$

Based on the findings of Zacharia [28,29,30], the radius of the spot size, *R_e_*, may be defined as the area covered by 95% of the laser power. This may be expressed as:

(6) $$\int_{0}^{2\pi }\int_{0}^{R_{e}}\frac{1}{2\pi s^{2}}e^{-\frac{r_{e}{}^{2}}{2s^{2}}}r_{e}dr_{e}d\theta =0.95.$$

The following relation may then be derived from Equation (6):

(7) $$s=\frac{R_{e}}{\sqrt{2\ln(20)}}\approx \frac{R_{e}}{\sqrt{6}},$$

By substituting Equation (7) into Equation (4), Equation (1) may be re-written as:

(8) $$E_{e}(r_{e})=\frac{3P_{i}}{\pi R_{e}{}^{2}}\exp(-\frac{3r_{e}{}^{2}}{R_{e}{}^{2}}),$$

As the vast majority of materials cannot fully absorb the energy provided by the laser, it is necessary to account for the laser energy absorption rate of the material used, *η_e_*. Based on the data provided by Dekock [31], *η_e_* was defined as 40%. The energy absorption of a material, *P_e_*, may then be expressed as:

(9) $$P_{e}=\eta_{e}\cdot \frac{3P_{i}}{\pi R_{e}{}^{2}}\exp(-\frac{3r_{e}{}^{2}}{R_{e}{}^{2}}),$$

The mathematical function for *P_e_* was written in the Fortran programming language. Using the subroutine flux interface provided by the boundary conditions function in MSC Marc, the numerical values calculated using the Fortran code were subsequently fed into MSC Marc to be used in subsequent calculations [32]. These calculations are as follows: In each time step, the Flux subroutine is called during each Gaussian integration point of the analysis with the appropriate flux type being specified in the DIST FLUXES input option, where the flux type is chosen according to the element type. The equivalent heat flux obtained from the mathematical function of *P_e_* at each node is then calculated and stored.

### 2.2. FE Model for Single-Track Laser Heat Treatment

**Figure 5:**
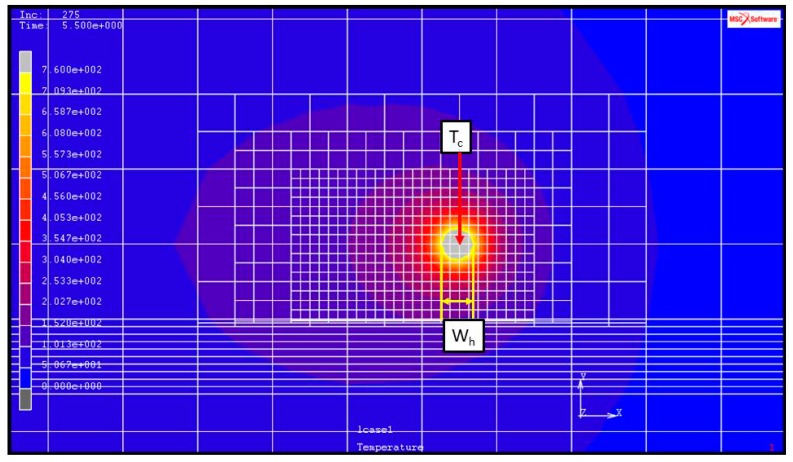
Example of the hardening width (*W_h_*) and maximum spot temperature (*T_c_*) caused by the application of a laser heat source.

**Figure 6:**
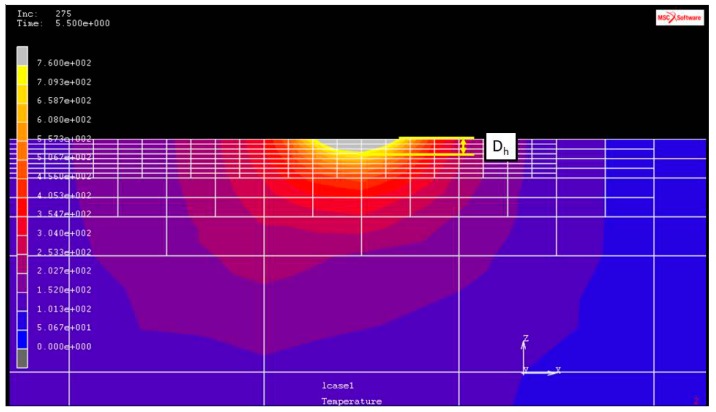
Example of the hardening depth (*D_h_*) caused by the application of a laser heat source.

The hardened layer width and depth determination is represented by a color matching temperature gradient, as shown in Figure 5 and Figure 6. The temperature is indicated above white at 760 °C represents that the temperature in this area has reached the quenching temperature. 

**Figure 7:**
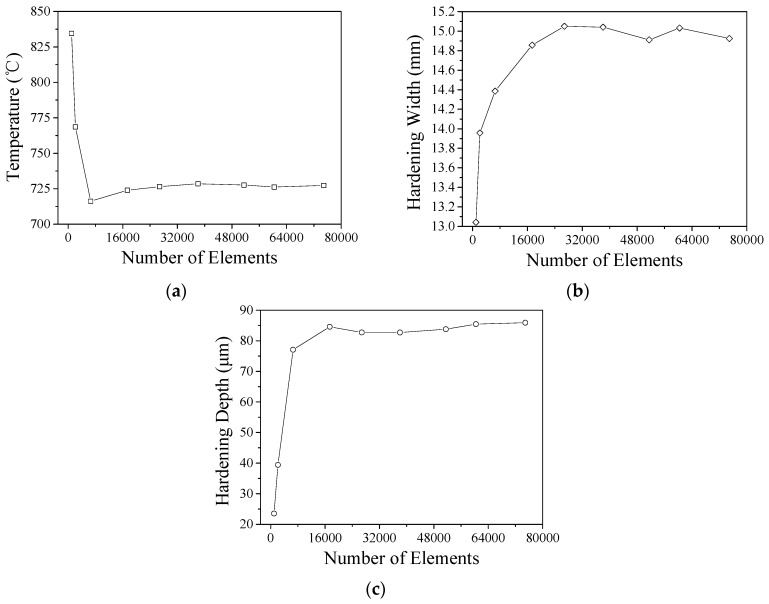
(**a**) Convergence analysis with temperature; (**b**) Convergence analysis with hardening width; and (**c**) Convergence analysis with hardening depth.

Due to the local heating of the material by YAG laser, the internal temperature gradient of the material is extremely narrow. During the calculation progress, when the analysis data diverges, the mesh will be partially refined. Using the element remesh to refine the heat affected zone range to improve data accuracy. The number of elements cannot be re-refined indefinitely. To increase the efficiency of the calculation, the mesh convergence analysis is shown in Figure 7. The results show that when the number of meshes reaches 17,000 or more, the values of the highest temperature, hardening width and depth of the hardened layer of the material are close to being stable. Therefore, the number of mesh cuts analyzed in the future is divided into more than 17,000 meshes.

The FE model that is being subjected to single-track laser heat treatment is shown in Figure 1. The heat treatment track is 80-mm long, and *P_s_* is the distance from the initial position of the heat treatment track. In this work, the maximum temperature of the laser spot, *T_c_*, was computed using the MSC Marc database for temperatures at the center of Gaussian lasers, as shown in Figure 1. Hardening widths and depths were deduced from the temperature distributions of the MSC Marc output files based on the previously defined heat treatment temperatures (760 °C for AISI 1045 and 850 °C for AISI 4140). The hardening widths and depths were then extracted and calculated in succession using geometric scale measurements. Figure 5 shows an example calculation of hardening width using this approach, while Figure 6 shows an example calculation of hardening depth. In the heat treatment of medium carbon steel, when the temperature reaches 760 degrees or more, it will rapidly cool, and the hardness of the surface of the material will increase greatly. Therefore, in this paper, the temperature distribution after finite element analysis is used to determine the region with a temperature higher than 760 degrees as the hardened region. The white area shown in Figure 5 and Figure 6 is the hardened area.

### 2.3. Experimental Setup

**Figure 8:**
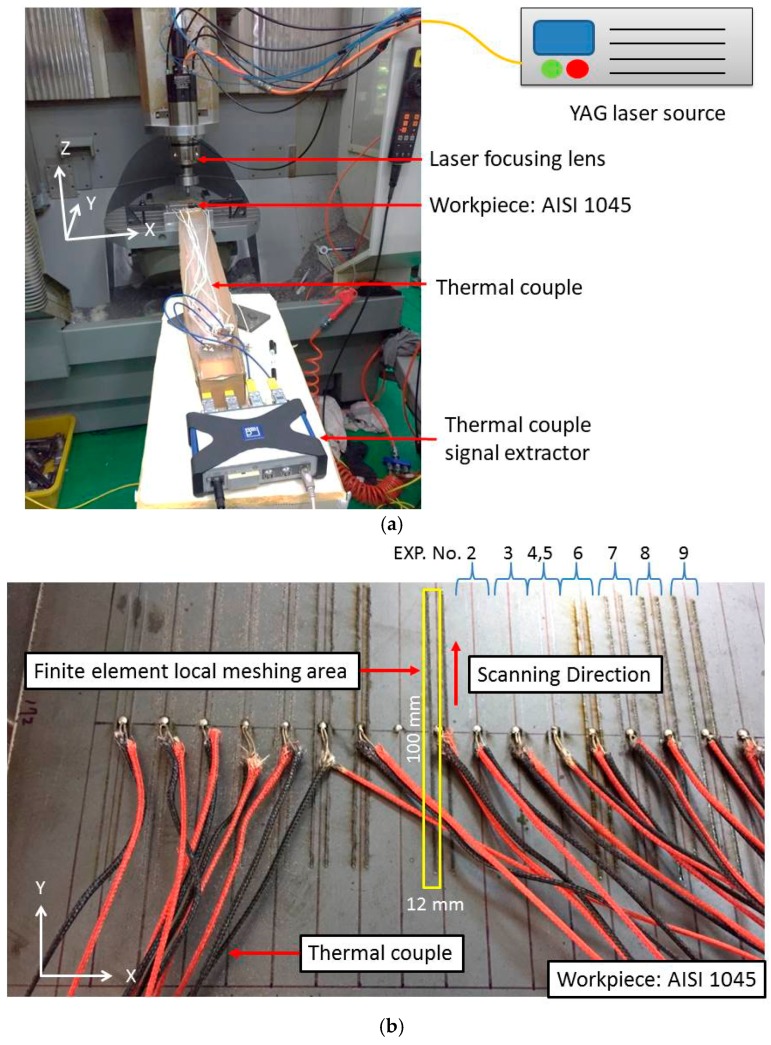
Experimental setup. (**a**) The arrangement of laser, workpiece and thermal couple; (**b**) Setup thermocouples on the workpiece and quenching tracks.

During the experiment, the laser lens and cooling water are set beside the CNC spindle, as shown in Figure 8a. A 1064-nm YAG laser with a maximum power of 1200 W was used in the experiment. During the laser scanning experiment, thermocouples were used to acquire the temperature data of the scanned tracks. The workpiece setup and thermocouples arrangement is shown in Figure 8b.

The dimensions of the AISI 1045 medium-carbon specimen used in this experiment were 210 mm × 170 mm × 6 mm. To simplify numerical calculations with different scanning speeds, various laser powers were used (ranging from 100 W up to 250 W), and the scanning speeds of the laser varied between 10 mm/s and 250 mm/s. The diameter of the laser spot was 1 mm. The thermocouple is welded at a distance of 1–1.5 mm from the center of the weld bead, as shown in Figure 8b. The Vickers hardness testing instruments was used to measure the surface hardness via laser heating during the hardness measurement experiment. 

## 3. Results and Discussion

### 3.1. Results of Finite Element Analysis and Experimental Results

**Figure 9:**
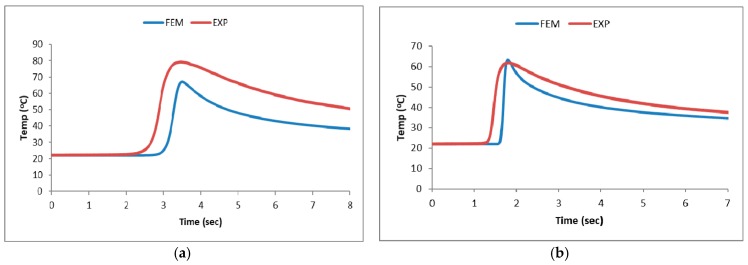

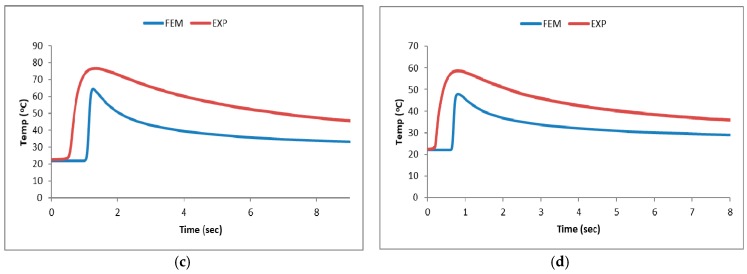
The temperature comparison of experimant ande FEA. (**a**) P = 100 W, Speed = 10 mm/s; (**b**) P = 100 W, Speed = 20 mm/s; (**c**) P = 250 W, Speed = 30 mm/s; and (**d**) P = 250 W, Speed = 50 mm/s.

The results obtained with the thermo-mechanical model proposed in this study for the analysis of laser quenching temperature fields was compared with our experimental results. We found that the predicted temperature variation curves are largely consistent with the experimental data. As there is a 1–1.5 mm gap between the measurement points and the hardening tracks, the measured temperature data actually corresponds to the surrounding temperature of the hardening tracks. Hence, the maximum measured temperatures will be slightly lower than the actual *T_c_*. This is clearly reflected in Figure 9. According to the temperature variation curves, temperature decreases as the scanning speed increases if the laser power is fixed. Therefore, we can infer that *T_c_* decreases as the scanning speed increases. In Figure 9, the main error in the finite element analysis and experimental temperature comparison is derived from the distance between the sampling point and the highest temperature. The distance on the finite element mesh model can be defined by the length of the grid and the highest temperature distance, fixed at 1.5 mm. It is extremely difficult to keep it fixed on the measurement. As the thermocouple has a 0.5 mm error with the centerline of the weld bead when welding the test piece. As the heat affected zone of the laser and the temperature distribution gradient is extremely narrow, small changes in the position of the thermocouple will affect the results of the comparison. 

From Table 2, it can be seen that the average hardness of the untreated material (No. 1) is 213 HV. In experiments No. 2, 4, 7, and 8, the surface hardness increased by more than 45%. Based on Experiments 8–12, increases in the scanning speed lead to decreases in surface hardness if the laser power was fixed. Similar results were obtained with other laser power-scanning speed combinations. Hence, laser power and scanning speed are the most important factors for determining quenching temperature. The surface hardness that were obtained using the aforementioned combinations of laser parameters are shown in Table 2.

### 3.2. Discussion of Parameters

The experimental results indicate that laser power and scanning speed are the most important factors for determining quenching temperature. In the following sections, the analytical model was used to perform an in-depth investigation on the effects of laser power, laser scanning speed, and laser spot size on the hardening thickness, hardening width, *T_c_*, and back-tempering effect on AISI 1045 medium-carbon steel and AISI 4140 alloy steel.

#### 3.2.1. Laser Power

**Figure 10:**
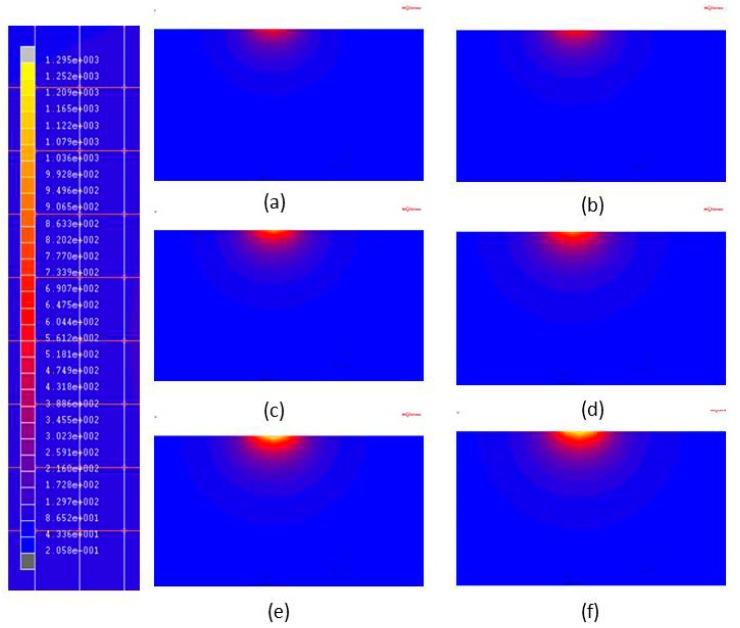
Temperature distribution profiles induced by different laser powers during single-track laser heat treatment. (**a**) AISI 1045, V_i_ = 8 mm/s, P_i_ = 250 W; (**b**) AISI 4140, V_i_ = 8 mm/s, P_i_ = 250 W; (**c**) AISI 1045, V_i_ = 8 mm/s, P_i_ = 350 W; (**d**) AISI 4140, V_i_ = 8 mm/s, P_i_ = 350 W; (**e**) AISI 1045, V_i_ = 8 mm/s, P_i_ = 450 W; and (**f**) AISI 4140, V_i_ = 8 mm/s, P_i_ = 450 W.

The laser hardening of AISI 1045 and AISI 4140 was simulated to observe the effects of using different laser powers on these systems. Three laser scanning speeds (1 mm/s, 8 mm/s and 16 mm/s) were used in these simulations, while the diameter of the laser spot was set to 3 mm. Figure 10 illustrates the laser-induced temperature distribution profiles in AISI 4140 and AISI 1045 steels for different laser powers and a laser scanning speed of 8 mm/s. The grey regions in these figures correspond to regions where melting occurred and it is shown that when Pi = 450 W, the melting zone is larger in AISI 1045 than in AISI 4140.

**Figure 11:**
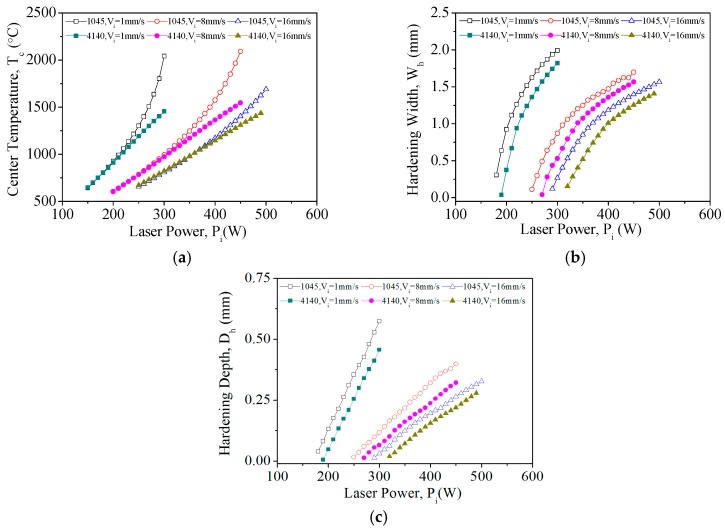
Single-track laser heat treatment. (**a**) Relationship between power and center temperature (*Tc*); (**b**) Relationship between laser power and hardening width; and (**c**) Relationship between hardening depth and laser power.

Given a constant laser scanning speed, the heat flux of each unit area increases with laser power, and the maximum spot temperature (*T_c_*) is also positively correlated with laser power. In Figure 11a, it is shown that Tc increases in proportion with laser power in AISI 1045 and AISI 4140 for all three scanning speeds. However, the temperature trends of each material are different: The temperature of AISI 1045 increases exponentially as the laser power increases, whereas the temperature of AISI 4140 increases linearly as the laser power increases. This could be caused by differences in the heat transfer coefficients of these materials. We then observed the temperatures of the materials when the laser power was varied between 300 W and 400 W for a scanning speed of 8 mm/s. Each additional 1 W of power increased the temperatures of AISI 1045 and AISI 4140 by 5.8 °C and 3.91 °C on average, respectively. Hence, the *T_c_* of AISI 1045 is more sensitive to increases in power compared with that of AISI 4140. In terms of the relationship between laser power and melting point, we found that AISI 1045 melted when the laser power ranged between 390 W and 410 W, while AISI 4140 melted when the laser power ranged between 410 W and 420 W.

We then observed the effects of laser power on the hardening width (*W_h_*) in AISI 4140 and AISI 1045. In Figure 11b, it is shown that the hardening widths of both materials increase with power in a non-linear quadratic fashion. When the laser scanning speed was 8 mm/s and the laser power was 350 W, hardening widths of 1.246 mm and 1.075 mm were obtained in AISI 1045 and AISI 4140, respectively. Hence, given the same processing conditions, AISI 1045 will yield a larger hardening width than AISI 4140; this difference could help to save energy. When the laser power was varied between 300 W and 400 W with a scanning speed of 8 mm/s, each additional 1 W of laser power increased the hardening widths of AISI 1045 and AISI 4140 by 0.06 mm and 0.083 mm, respectively. This indicates that AISI 4140 is more sensitive to increases in power than AISI 1045 in terms of the hardening width when the same laser scanning speed is used. 

Finally, we inspected the effects of laser power on hardening depth (*D_h_*) in AISI 1045 and AISI 4140. In Figure 11c, it is shown that the hardening depths of both materials increases linearly with laser power. When the power of the laser was set to 350 W at a scanning speed of 8 mm/s, we found that the corresponding hardening depths of AISI 1045 and AISI 4140 were 0.217 mm and 0.161 mm, respectively. In other words, provided the same processing conditions are met, AISI 1045 will have a greater hardening depth than AISI 4140, thus making the former better suited for mechanical parts that undergo long periods of wear. When the laser power was varied between 300 W and 400 W with a scanning speed of 8 mm/s, we found that each additional 1 W of power increased the hardening depths of AISI 1045 and AISI 4140 by 0.02 mm and 0.017 mm, respectively, which is the opposite of the trend that was observed for the hardening width. Hence, given the same laser scanning speed, AISI 1045 is more sensitive to increases in laser power than AISI 4140 in terms of hardening depth.

#### 3.2.2. Laser Scanning Speed

**Figure 12:**
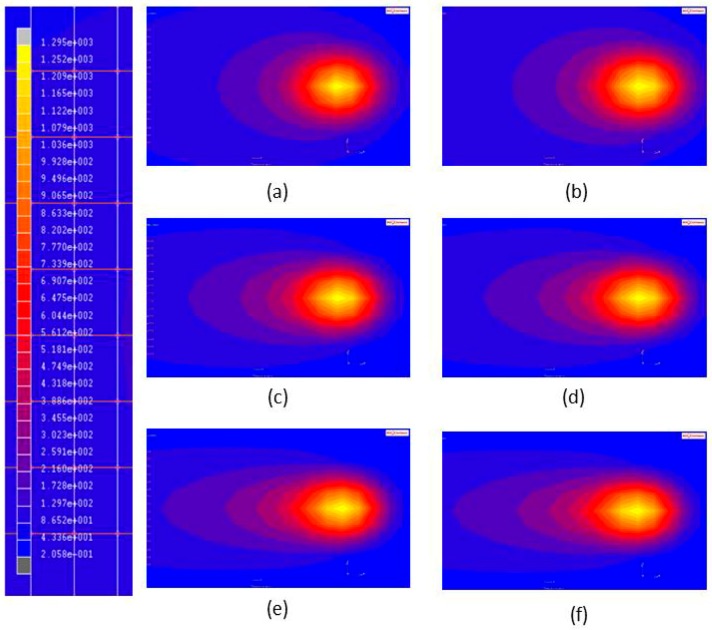
Temperature distributions induced by different scanning speeds during single-track laser heat treatment. (**a**) AISI 1045, V_i_ = 10 mm/s, P_i_ = 350 W; (**b**) AISI 4140, V_i_ = 10 mm/s, P_i_ = 350 W; (**c**) AISI 1045, V_i_ = 25 mm/s, P_i_ = 350 W; (**d**) AISI 4140, V_i_ = 25 mm/s, P_i_ = 350 W; (**e**) AISI 1045, V_i_ = 40 mm/s, P_i_ = 350 W; and (**f**) AISI 4140, V_i_ = 40 mm/s, P_i_ = 350 W.

The effects of laser scanning speed on steel materials were simulated and the results are presented in this section. The laser powers used in these simulations were 200 W, 350 W, and 500 W, while the spot diameter was set to 3 mm. The temperature distributions of both aforementioned steels, when a laser power of 350 W was used, are shown in Figure 12. In that figure, it is shown that increases in scanning speed exacerbate the hysteresis of the temperature field distribution and narrow the width of the temperature field.

**Figure 13:**
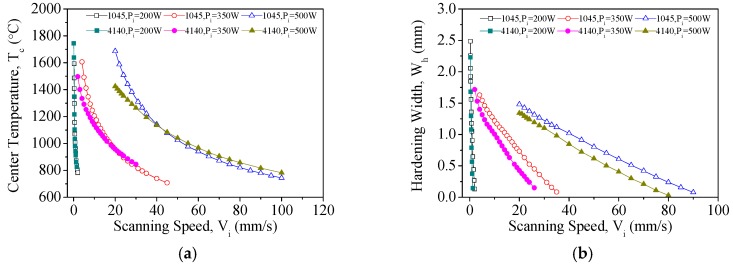

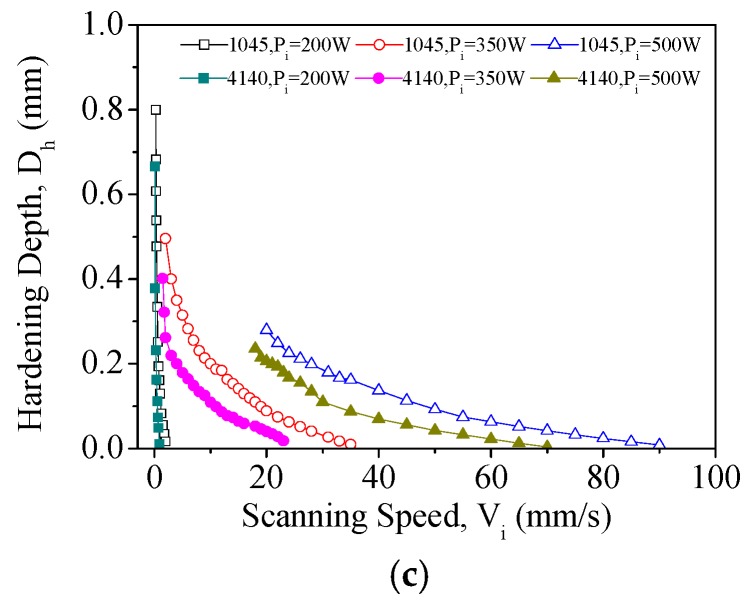
(**a**) Relationship between scanning speed and temperature during single-track laser heat treatment; (**b**) relationship between scanning speed and hardening width during single-track laser heat treatment; and (**c**) relationship between scanning speed and hardening depth during single-track laser heat treatment.

Given the same laser power, an increase in scanning speed decreases the heat flux received by a unit area in each unit of time; hence, the temperature of the material should correlate negatively with scanning speed. In Figure 13a, it is shown that a very large range of laser scanning speeds (*V_i_*) is required to maintain *T_c_* within the range of heat treatment temperatures for AISI 1045 and AISI 4140 with the three aforementioned laser powers. In AISI 1045, scanning speeds of 0.25 mm/s and 1.25 mm/s led to temperatures of 1593 °C and 873 °C, respectively, when the laser power (*P_i_*) was 200 W. Given a laser power of 500 W, *T_c_* was 1588 °C when the scanning speed was 22 mm/s, but the latter had to be increased up to 70 mm/s to produce a *T_c_* of 873 °C. Similar trends were observed for AISI 4140. Hence, in the planning of laser heat treatment parameters, laser power plays an important role in determining the maximum temperatures of the heat treatment process and also the laser scanning speeds that are necessary for this process. We then observed the temperatures of AISI 1045 and AISI 4140 when the laser scanning speed was varied between 10 mm/s and 20 mm/s with a laser power of 350 W. We found that each additional 1 mm/s of scanning speed decreased the temperature of AISI 1045 and AISI 4140 by an average of 24.8 °C and 18.1 °C, respectively. AISI 1045 is therefore more sensitive to scanning speed than AISI 4140 in terms of *T_c_*.

In the next step, we observed the effects of laser scanning speed on hardening width (*W_h_*). In Figure 13b, it can be seen that the hardening width of both materials decreased quadratically as the scanning speed increased. Given a laser power of 350 W, a hardening width of 1 mm can be achieved in AISI 1045 with a scanning speed of 15 mm/s, whereas a scanning speed of 10 mm/s is required to achieve the same hardening width in AISI 4140. Therefore, the same hardening width can be achieved more quickly in AISI 1045 than in AISI 4140, which significantly shortens processing times. Finally, we observed the changes in hardening width that accompanies the variation of scanning speed between 10 mm/s and 20 mm/s with a laser power of 350 W. Each additional 1 mm/s in scanning speed reduced the hardening widths of AISI 1045 and AISI 4140 by 0.048 mm and 0.058 mm on average, respectively. Hence, given the same laser power, AISI 4140 is more sensitive to scanning speed than AISI 1045 in terms of hardening width.

We then observed the effects of laser scanning speed on the hardening depths (*D_h_*) of these materials. In Figure 13c, it is shown that the hardening depth of both materials decreases logarithmically as the laser scanning speed increases. It is also shown that the same hardening depth can be achieved with higher scanning speeds in AISI 1045 than in AISI 4140 (using the same laser power). Based on the data for AISI 1045 and AISI 4140 that was obtained using three different laser powers, we found that greater hardening depths can be achieved with lower laser powers and scanning speeds if the hardening process is constrained to temperatures for which material melting does not occur. In AISI 1045, a laser power of 200 W with a scanning speed of 0.28125 mm/s leads to a *T_c_* of 1487 °C and a *D_h_* of 0.683 mm, while a laser power of 350 W and a scanning speed of 5 mm/s produces a *T_c_* of 1492 °C and a *D_h_* of 0.315 mm; the hardening depth of the latter is less than half of the former. When the scanning speed of the laser was varied between 10 mm/s and 20 mm/s with a power of 350 W, each additional 1 mm/s in scanning speed decreased the hardening depths of AISI 1045 and AISI 4140 by 0.011 mm and 0.008 mm, respectively. AISI 1045 is therefore more sensitive to laser scanning speed than AISI 4140—in terms of hardening depth. 

**Figure 14:**
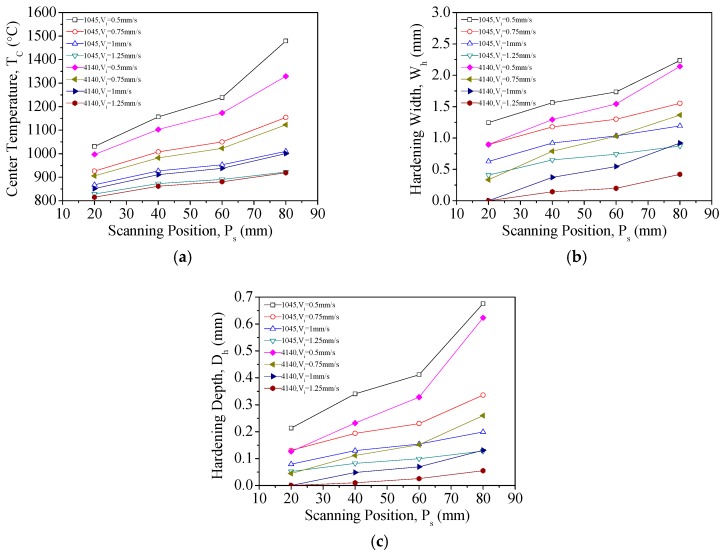
(**a**) Relationship between temperature and scanning position for a laser power of *P_i_* = 200 W; (**b**) relationship between hardening width and scanning position for a laser power of *P_i_* = 200 W; and (**c**) relationship between hardening thickness and scanning position for a laser power of *P_i_* = 200 W.

Figure 14a–c describe how scanning position (*P_s_*) is related to temperature, hardening width, and hardening depth, respectively. In Figure 14a, it is shown that heterogeneities in temperature occurs with changes in scanning position in both materials when the laser power is 200 W and low scanning speeds are used. When the scanning speed was 0.5 mm/s, the *T_c_* of AISI 1045 at *P_s_* = 80 mm was 43% higher than the corresponding *T_c_* at *P_s_* = 20 mm. In AISI 4140, the *T_c_* at *P_s_* = 80 mm was higher than that at *P_s_* = 20 mm by 33%; heterogeneities in temperature are therefore quite pronounced in these cases. Upon further inspection, Figure 14b,c shows that the *W_h_* and *D_h_* of the materials become heterogeneous with changes in the scanning position. This may be caused by low scanning speeds, which leads to large accumulations of energy that cannot be dissipated via heat transfer or convection. Hence, low laser power and low scanning speed is the worst possible combination of laser heat treatment parameters in terms of temperature uniformity.

#### 3.2.3. Laser Spot Size

**Figure 15:**
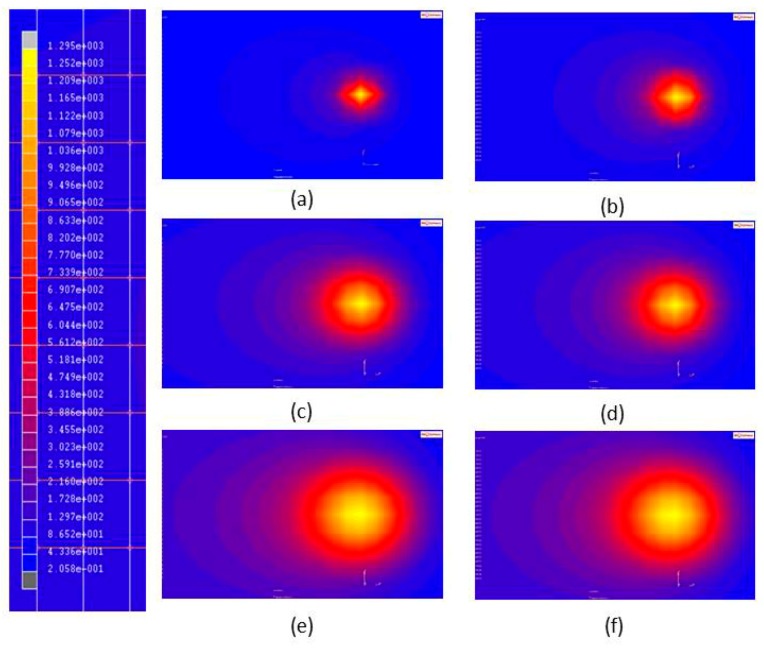
Temperature distributions induced by different laser spot sizes during single-track laser heat treatment. (**a**) AISI 1045, R_i_ = 2 mm; (**b**) AISI 4140, R_i_ = 2 mm; (**c**) AISI 1045, R_i_ = 3 mm; (**d**) AISI 4140, R_i_ = 3 mm; (**e**) AISI 1045, R_i_ = 4 mm; and (**f**) AISI 4140, R_i_ = 4 mm.

The effects of laser spot size on the maximum temperature (*T_c_*), hardening width (*W_h_*), and hardening depth (*D_h_*) of the aforementioned steels were predicted using the FE model. The parameters of the laser heat treatment were a laser power of 350 W and a scanning speed of 8 mm/s, while the diameter of the laser spot (*R_i_*) was varied between 2 mm and 4 mm. The temperature distributions in the two materials that were generated using three different laser spot sizes are shown in Figure 15. 

**Figure 16:**
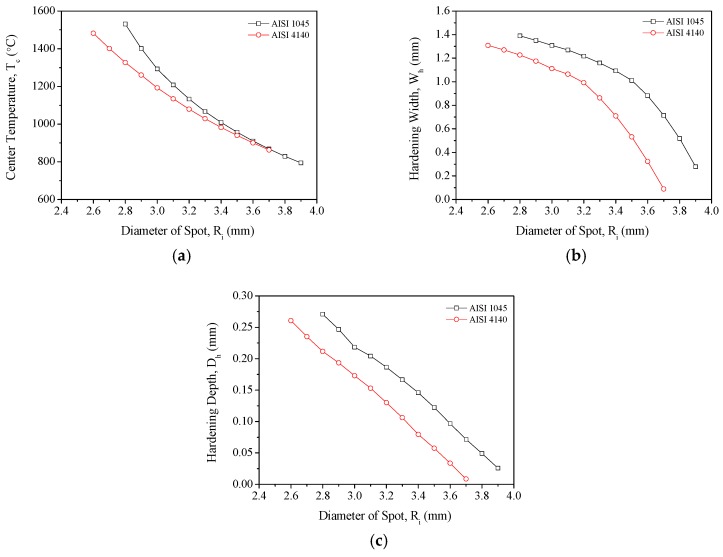
(**a**) Relationship between laser spot size and *T_c_* during single-track laser heat treatment; (**b**) relationship between laser spot size and hardening width during single-track laser heat treatment; and (**c**) relationship between hardening width and laser spot size during single-track laser heat treatment.

Given the same laser power and scanning speed, it can be inferred from the Gaussian distribution presented in Equation (9) that the heat flux received by each unit area of the surface is determined by the size of the laser spot. Furthermore, the relationship between laser spot size and heat flux is not linear. In Figure 16a, it is shown that the laser-induced *T_c_* of AISI 1045 and AISI 4140 decreases quadratically as *R_i_* increases. We then observed that *T_c_* induced by spot sizes ranging between 2.5 mm and 3.5 mm; for each 0.1-mm increase in spot size, the *T_c_* values of AISI 1045 and AISI 4140 decreased by 137.4 °C and 63 °C on average, respectively. Hence, AISI 1045 is more sensitive than AISI 4140 to increases in laser spot size in terms of *T_c_*. By observing the relationship between laser spot size and material melting, we found that melting occurs in AISI 1045 for spot sizes between 2.8 mm and 2.9 mm, while AISI 4140 melts when the spot size ranges from 2.6 mm to 2.7 mm.

The effects of laser spot size on hardening width (*W_h_*) were then observed. In Figure 16b, it is shown that hardening width decreases quadratically as the laser spot size increases because the power density was decreased in both AISI 4140 and AISI 1045. The hardening widths that were generated using laser spot sizes between 2.5 mm and 3.5 mm were then observed. We found that each 0.1-mm increase in laser spot size decreased the hardening widths of AISI 1045 and AISI 4140 by 0.043 mm and 0.081 mm on average, respectively. This shows that the hardening widths of AISI 4140 are more sensitive to changes in laser spot size compared with those of AISI 1045. The data also shows that the *T_c_* values associated with a laser spot size of 2.5 mm either reach or exceed the melting points of both materials. Nonetheless, even when the spot size was only 2.5 mm, the hardening width-to-laser spot size ratio of AISI 1045 and AISI 4140 were only 0.57 and 0.53, respectively, which are lower than the ideal ratio of 0.7.

Finally, we observed the effects of laser spot size on hardening depth (*D_h_*). In Figure 16c, it is shown that hardening depth decreases linearly as the laser spot size increases. Based on the hardening depths associated with laser spot sizes between 2.5 mm and 3.5 mm, we found that each 0.1-mm increase in laser spot size decreased the hardening depths of AISI 1045 and AISI 4140 by 0.021 mm and 0.023 mm, respectively. AISI 4140 therefore has a greater degree of sensitivity to changes in laser spot size than AISI 1045 in terms of hardening depth.

The results of FE simulations are summarized in Table 3 and Table 4. Among the combination of parameters that induce the quenching effect, we found that changes in laser power, scanning speed, and laser spot size result in significant changes in material temperature, hardening width, and hardening depth. The data shown in Table 3 and Table 4 can be used to plan laser processing parameters or to predict the thickness and width of the hardened layer after a laser quenching process. Under certain processing parameters, the laser spot diameter increases or increases the scanning speed. The hardening depth, width, and maximum laser spot temperature of the material will decrease as shown in Table 3. Table 4 shows that increase in the laser power at a fixed scanning speed and spot size to get deeper hardening depth and range. 

## 4. Conclusions

In this paper, a finite element analysis model of laser quenching heat treatment is proposed, which can analyze a laser heat affected zone, hardening area after quenching, and temperature variation during the laser heating process. Using the analysis data, it is possible to predict whether the processing parameters are sufficient to achieve the quench effect. Experiments were performed to measure the changes in temperature associated with a moving laser, and the results of these experiments were compared to those of a numerical analysis model proposed in this study. We found that the temperature trends predicted using the FE model is in close agreement with the experimental data, thus validating the accuracy of FE model. The numerical model was used to investigate the effects of various laser parameters (spot size, scanning speed, scanning position, and laser power) on the maximum temperature (*T_c_*), hardening width (*W_h_*), and hardening depth (*D_h_*) of AISI 1045 and AISI 4140 steels during single-track laser heat treatment using an Nd:YAG laser. The proposed FE model can also predict the hardening area of the material by adjusting the laser spot size, laser power, and scanning speed. The analysis results indicate that increasing the laser power will increase the hardening width and depth at a defined scanning speed and laser diameter. In specific cases, increasing the laser diameter and scanning speed will reduce the hardening width and depth.

## Figures and Tables

**Table 1:** Basic material properties.

Property	AISI 1045	AISI 4140
Density (Kg/m^3^)	7870	7850
Thermal Conductivity (W/m·°C)	Figure 2	Figure 3
Specific Heat (J/Kg·°C)	Figure 2	Figure 3
Young’s Modulus (GPa)	Figure 2	Figure 3
Yield Strength (MPa)	310	415
CTE (Coefficient of Thermal Expension) (μm/m·°C)	15	15
Poisson’s Ratio	0.27	0.3
Hardening Temperature $T_{h}$ (°C)	760	850
Melting Temperature $T_{m}$ (°C)	1520	1410
Tempering Temperature $T_{t}$ (°C)	400	400

**Table 2:** Hardnesses obtained for different combinations of laser parameters.

No.	Laser Power (W)	Laser Scanning Speed (mm/s)	Average Hardness (HV)	Increase in Hardness (%)
1	0	0	213	0
2	100	10	472	55
3	100	20	303	30
4	125	10	390	45
5	125	30	286	26
6	200	30	286	26
7	200	50	385	45
8	250	30	397	46
9	250	50	301	29
10	250	100	298	29
11	250	200	297	28
12	250	250	269	21

**Table 3:** Recommended processing parameters to reduce hardening zone

**Processing Parameters**	**Increasing Laser Spot Diameter by 0.1 mm**			**Increasing Laser Scanning Speed by 1 mm/s**		
	Laser Spot Diameter Range: 2.5–3.5 mm Laser Power: 350W Scanning Speed: 8 mm/s			Scanning Speed Range: 10–20 mm/s Laser Power: 350W Laser Spot Diameter: 3 mm		
**Materials**	**Temperature (K)**	***W_h_* (mm)**	***D_h_*** **(mm)**	**Temperature (K)**	***W_h_* (mm)**	***D_h_*** **(mm)**
AISI 1045	−137.4	−0.043	−0.021	−24.8	−0.048	−0.011
AISI 4140	−63	−0.081	−0.023	−18.1	−0.058	−0.008

**Table 4:** Recommended processing parameters to increase hardening zone.

**Processing Parameters**	**Increasing Laser Power by 1 W**		
	Laser Power Range: 300–400W Scanning Speed: 8 mm/s Laser Spot Diameter: 3 mm		
**Materials**	**Temperature (K)**	***W_h_* (mm)**	***D_h_*** **(mm)**
AISI 1045	+5.8	+0.06	+0.02
AISI 4140	+3.91	+0.083	+0.017
